# A Genome-Wide Analysis of *StTGA* Genes Reveals the Critical Role in Enhanced Bacterial Wilt Tolerance in Potato During *Ralstonia solanacearum* Infection

**DOI:** 10.3389/fgene.2022.894844

**Published:** 2022-07-26

**Authors:** Tian Tian, Ruimin Yu, Yanyun Suo, Lixiang Cheng, Guizhi Li, Dan Yao, Yanjie Song, Huanjun Wang, Xinyu Li, Gang Gao

**Affiliations:** College of Life Sciences, Shanxi Normal University, Taiyuan, China

**Keywords:** potato, *StTGA*, qRT-PCR, MDA, Y1H

## Abstract

*TGA* is one of the members of TGACG sequence-specific binding protein family, which plays a crucial role in the regulated course of hormone synthesis as a stress-responsive transcription factor (TF). Little is known, however, about its implication in response to bacterial wilt disease in potato (*Solanum tuberosum*) caused by *Ralstonia solanacearum*. Here, we performed an *in silico* identification and analysis of the members of the *TGA* family based on the whole genome data of potato. In total, 42 *StTGAs* were predicted to be distributed on four chromosomes in potato genome. Phylogenetic analysis showed that the proteins of *StTGAs* could be divided into six sub-families. We found that many of these genes have more than one exon according to the conserved motif and gene structure analysis. The heat map inferred that *StTGAs* are generally expressed in different tissues which are at different stages of development. Genomic collinear analysis showed that there are homologous relationships among potato, tomato, pepper, Arabidopsis, and tobacco *TGA* genes. Cis-element *in silico* analysis predicted that there may be many cis-acting elements related to abiotic and biotic stress upstream of *StTGA* promoter including plant hormone response elements. A representative member *StTGA39* was selected to investigate the potential function of the *StTGA* genes for further analysis. Quantitative real-time polymerase chain reaction (qRT-PCR) assays indicated that the expression of the *StTGAs* was significantly induced by *R. solanacearum* infection and upregulated by exogenous salicylic acid (SA), abscisic acid (ABA), gibberellin 3 (GA_3_), and methyl jasmonate (MeJA). The results of yeast one-hybrid (Y1H) assay showed that *StTGA39* regulates *S. tuberosum* BRI1-associated receptor kinase 1 (*StBAK1*) expression. Thus, our study provides a theoretical basis for further research of the molecular mechanism of the *StTGA* gene of potato tolerance to bacterial wilt.

## Introduction

Plants face a variety of challenges and stresses during growth and development. Specific transcription factors (TF) in plants can specifically regulate the expression of plant genes to enhance the ability of plants to adapt to stress environment. In plants, the gene regulatory network which TFs participate in plays a crucial role in the stress response pathway (Li B. et al., 2019). The members of the *basic Region-Leucine Zipper* (*bZIP*) gene family are crucial regulators, which play an important role in development, stress adaptation, and hormone synthesis of plants (Azeem et al., 2020; Kumar et al., 2021). The TGACG sequence-specific binding protein family (TGA) TFs belong to a subfamily of bZIP and play an important role in abiotic stress responses during the period of plant growth (Ke et al., 2022). The TGA TFs include large regulatory regions, which may affect DNA-binding by allosteric or electrostatic interaction as for the bZIP (Salladini et al., 2020).

Some *TGA* members play crucial roles in stress mitigation. Evidence revealed that the role of *TGA* class II is important during the response of Arabidopsis to control reactive oxygen species (ROS) levels (Ariel et al., 2021). In addition, there was evidence that Cr^6+^ can cause the binding of TGA3 to the L-cysteine desulfhydrase (LCD) promoter in plants, thus increasing the expression of LCD (Fang et al., 2017). Furthermore, glutaredoxins (GRXs) may play a crucial role in the formation of floral organs with *TGA* (Rouhier et al., 2015). ROXYs (GRX,CC-type glutaredoxin, named ROXYs in *Arabidopsis thaliana*) and CC-type GRXs from rice and corn interact with the TGA family to control developmental processes (Gutsche et al., 2017; Uhrig et al., 2017).

An important role of *TGAs* during plant development has been covered in a variety of plants. It can be seen that the *TGA* gene family participates in many signaling pathways in plants and plays a crucial role during plant growth. In soybean, the *TGA* plays a crucial role in response to nitrogen availability (Ullah et al., 2019). Biosynthesis of salicylic acid (SA) is indirectly modulated by TGA1 and TGA4 TF (Budimir, et al., 2020). Nonexpresser of pathogenesis-related genes (NPR1) recruits TGA TFs in the presence of SA and facilitates gene expression to establish plant immunity (Chen et al., 2019). Multiple TGA members have been shown to play a key role in plant immunity. *Oryza sativa* TGA2 (OsTGA2) can directly regulate defense-related genes (Moon et al., 2018). The key points in the regulation of sunflower resistance by *TGA* may be involved in the resistance of sunflower to *Verticillium dahliae* (Guo et al., 2017). In addition, ROXY can be used as TGA-dependent promoter to control the negative regulators of detoxification genes in *A. thaliana* (Huang et al., 2016). Brassinoidsteroid (BR) induces apoplastic ROS, which activates the TGA2 factor and triggers the metabolism of pesticide residues in tomato (Hou et al., 2019). Taken together, studies of TGA TFs and the TGA family as a whole demonstrate that TGA proteins play important roles in regulating multiple biological processes in plants.

Although there are many studies on *TGA*, no study has been reported in potato (*Solanum tuberosum*), which is an important food crop and widely cultivated and consumed all over the world (Gebhardt, 2016). There are many kinds of major diseases affecting potato production worldwide, and bacterial wilt caused by *Ralstonia* *solanacearum* is the second one in importance (Barchenger et al., 2022). *R. solanacearum* is a soil-borne, devastating plant pathogen and uses type III effectors to inhibit the plant immune system (Salanoubat et al., 2002; Qi et al., 2022). This pathogen has a remarkably wide host range and global distribution and is involved in the invasion of plant root to the vessel of xylem and may eventually lead to plant death (Gutsche and Zachgo, 2016; Ferreira et al., 2017).

In the current study, a comprehensive investigation of TGA TFs in potato was conducted, and the analysis of the member distribution, evolutionary model, gene structure, and expression patterns was performed. In addition, we found a representative member *S. tuberosum TGA39 (StTGA39)* that could be induced by abiotic/biotic stress and has a critical role in enhanced bacterial wilt tolerance in potato during *R. solanacearum* infection. This will lay a foundation for further research on the function of *StTGA* genes.

## Materials and Methods

### Identification and Classification of *StTGA* Gene Family

*StTGA* gene identification in the potato genome was performed using BLAST and hidden Markov models (HMM) search methods (Wang et al., 2018). We downloaded the potato genome from the Phytozome database. Briefly, the HMM seed file of delay of germination 1 (DOG1) domain (accession number: PF14144), which belongs to TGA, was downloaded from the Pfam database (http://pfam.xfam.org/). A round of HMM scan was performed for all the obtained hits against the Pfam database. We used the HMMER program to search all putative TGA protein sequences and extracted the corresponding sequence IDs, with expectation value (E-value) set to 1.0. The sequences IDs were submitted to the Spud DB (http://solanaceae.plantbiology.msu.edu/). The results from the two methods were compared, and common sequence IDs were selected. In total, 42 members of the *StTGA* gene family in potato were predicted and named *StTGA01–StTGA42*. The information about the renaming *StTGA* genes in potato is provided in Supplementary Additional File S1. The physicochemical parameters, such as molecular weight and isoelectric points (pIs), and subcellular localization of each StTGA protein were predicted *via* ExPASy (http://web.expasy.org/protparam/) and PSORT (https://wolfpsort.hgc.jp/) online tools (Liang et al., 2017).

### Phylogenetic Analysis, Conserved Motif Analysis, *In Silico* Chromosome Mapping, and Gene Structure Analysis of *StTGA*

We constructed the phylogenetic tree by MEGA 7.0 *via* the neighbor-joining (NJ) method, using 1000 bootstrap iterations and default parameters (Gao et al., 2018). The potato gff3. annotation file was parsed to extract the genome locations of the identified *TGA* genes. According to this information, we visualized the *in silico* predicted chromosomal distribution of *StTGA* genes by TBtools software (Chen et al., 2020). The *in silico* exon–intron distribution of *StTGA* genes was predicted through the Gene Structure Display Server (GSDS) website (http://gsds.gao-lab.org/index.php). Conserved motifs of *StTGA* genes were predicted by the online service Multiple Em for Motif Elicitation (MEME) (Fan et al., 2021a). The information of *in silico* chromosome location of *StTGA* was retrieved from potato genome data downloaded from the phytozome database (Chen et al., 2020). The secondary and 3D structure prediction of StTGA proteins was performed using SOPMA and I-TASSER web servers, and the data were captured using Discovery Studio 4.5 (Geourjon and Deléage, 1995; Ambrish et al., 2010). The upstream 2000 bp region of the initiation codon ATG of each *StTGA* gene was used to search the promoter region in the downloaded sequence and predicted potential cis-acting elements of each binding site on PlantCARE website (Lescot et al., 2002; Zhu et al., 2020).

### Collinearity Analysis of *StTGAs*

Pair-wise all-against-all BLAST was performed for potato, tomato, tobacco, Arabidopsis, and pepper protein sequences. The obtained results and the gff3. annotation file were then determined by the Multiple Collinearity Scan toolkit (MCScanX) for determination of the gene duplication type (Wang et al., 2012; Fan et al., 2021b). Microsynteny relationships between potato and the other four species were analyzed to show the gene homology relationship (Gutsche and Zachgo, 2016), and visualized by TBtools (Chen et al., 2020).

### RNA-Seq Analysis of *StTGAs*

Based on the digital expression RNA-Seq data retrieved by previous methods (Cao et al., 2021), the expression level of *StTGAs* in different tissues and developmental stages under abiotic/biotic stress was indicated using Fragments Per Kilobase of transcript sequence per Millions base pairs (FPKM), which were retrieved through the website Spud DB (http://solanaceae.plantbiology.msu.edu/) (Cao et al., 2021). The heatmaps of *StTGA* expression and hierarchical clustering analysis were conducted using the TBtools software.

Furthermore, the co-expression pattern of *StTGA* genes was analyzed based on the Pearson correlation coefficient (PCC) and graphically presented using the Cytoscape package. The PCC and mutual rank (MR) were calculated according to previous reports (Da et al., 2019). Forty-two potato StTGA differently expressed genes and 24 related genes were analyzed by co-expression network analysis (Renamed in Supplementary Additional File S2). The interaction network of differentially expressed TFs was built by STRING: functional protein association networks (https://string-db.org/) and determined by Cytoscape (Kohl et al., 2011; He et al., 2022). When the PCC was greater than 0.8, it was assumed that they were co-expressed.

### Plant Material and Growth Conditions

Potato plants (ZHONG 3,2 *n* = 48, tetraploid cultivation) used in this study were provided by the Chinese Academy of Agricultural Sciences (CAAS). The pots which were used to culture these plants were 10 cm in diameter and 15 cm in height, and they contained 450 ml peat and vermiculite (3:1, volume ratio). In the process of culture, *S. tuberosum* (ZHONG 3) wild-type plants were grown under long-day conditions (16 h, 26°C, day/8 h, 18°C, night) on the abovementioned mixed soil. The light intensity was about 3000 lx, and the relative humidity was 75% (Gao et al., 2010; Yu et al., 2021a).

### Bacterial Strains and Inoculum

*R. solanacearum* belongs to PO41 strain (seed type II, race 3 biotype 2) (He et al., 1983), which was cultured in a CPG medium, and then the solution was diluted to 10^8^ cfu/ml (Optical Density, OD600 = 0.2). Potatoes were inoculated by the root injury irrigation method when they grew to the stage of two-week-old potato plants with 7–8 leaves (Yu et al., 2021b). The control group was inoculated with the same amount of water. At 12, 24, 36, 48, 60, 72, 84, 96, 108, and 120 hpi after inoculation, we sampled the leaves of the experimental group and control group. All samples were frozen in liquid nitrogen immediately and then stored at −80°C (Kong et al., 2016). Three replicates were performed for both *R. solanacearum* and mock inoculation.

### Hormone Treatment

When potatoes grew to the stage of two weeks with 7–8 leaves, the leaves were sprayed with 100 μM abscisic acid (ABA), 350 μM, gibberellin 3 (GA_3_), 50 μM SA, and 50 μM methyl jasmonate (MeJA) (Yu et al., 2021a). At 1, 2, 3, 4, and 5 dpi, we sampled the leaves of each hormone treatment. All samples were frozen in liquid nitrogen immediately and then stored at −80°C until these samples were used to extract RNA (Fan et al., 2021a). Each treatment was repeated three times.

### Verification of RNA-Seq Data

The RNA-seq data of the *StTGA39* gene were verified by qRT-PCR according to the methods of Schmittgen and Livak, 2008. The leaf samples for RNA extraction were previously stored at −80°C, and the total RNA was isolated using the TRIzol reagent (Invitrogen, Carlsbad, CA, United States) according to the manufacturer’s protocol. The mRNA was re-transcribed into cDNA using the Prime Script cDNA Synthesis Kit (TransGen, Beijing, China). Then, cDNA was used as a template for qRT-PCR, which was performed on the Quant Studio 3 qRT-PCR System (Thermo Fisher Scientific, Shanghai, China) (Kong et al., 2016). The following primers were used in the process of qRT-PCR with three replicates for each gene as described previously: StTGA-FP: TCC​AGC​ACA​TCC​AAC​ACC; StTGA-RP: TTC​ACC​AAG​ATT​TCC​CAC. ACTIN of potato served as the internal control (GenBank Accession: X55747), and the following primers were used in the process of qRT-PCR with three replicates for each gene as described previously: Actin-F: TAT​AAC​GAG​CTT​CGT​GTT​GCA​C; Actin-R: ACT​GGC​ATA​CAG​CGA​AAG​AAC​A.

### Staining Treatment and Quantifying Levels of Malondialdehyde

After the potato seedlings were inoculated with *R. solanacearum* or water for 72 hpi, the leaves were collected for observing the infection of *R. solanacearum*. The leaves were left in trypan blue solution for 24 h (Yu et al., 2021b). Levels of H_2_O_2_ were detected using the DAB staining method by the DAB Substrate Kit (Solarbio, Beijing, China). Leaves were soaked in the solution to stain for 3–10 min under dark conditions (Christensen et al., 1997; Song et al., 2021). The leaves were sampled from inoculated plants by *R. solanacearum* at 12, 24, 36, 48, 60, 72, 84, 96, 108, and 120 hpi for quantifying levels of MDA. The content of MDA of these leaves was tested by using an MDA Assay Kit (Abbkine, Wuhan, China) according to the previous method (Islamoglu et al., 2018). OD at 532 and 600 nm was determined by a microplate reader.

### Y1H Assays

In order to analyze whether there is a TGACG cis-element in the upstream promoter sequence of the gene homologous to *S. tuberosum Brassinosteroid insensitive 1 (BRI1)-associated receptor kinase 1 (StBAK1)*, we analyzed the relationship between StTGA1 and *StBAKl* by the Y1H assay Kit (Clontech, State of California, United States) in this study. Briefly, the sequence of *StBAK1* with 1512 bp was amplified from *S. tuberosum* cDNA by gene-specific primers and digested by EcoRI (CAATTC) and MluI (ACGCGT) so that the oligonucleotide sequences containing TGA cis-elements and other corresponding promoter sequences were cloned into the pHIS2 to generate the reporter vector. All of the constructs were co-transformed into yeast strain Y187 according to the manufacturer’s instructions (Liu et al., 2016). The full-length open-reading frame (ORF) of StTGA (855bp) was fused to the GAL4 activation domain in the vector pGADT7 digested with EcoRI (CAATTC) and BamHI (GGATCC) to get a fusion protein TGA-pGADT7 (effector vector) (Huang et al., 2010). The others were carried out following the manufacturer’s instructions.

### Statistical Analysis

Standard values and standard error of experimental data were calculated using Microsoft Excel 2016 and *n* = 3 for independent experiments according to a *t*-test. Analyses of the significance of differences were conducted *via* the data processing System. The PCC is calculated by the SPSS online services tool (https://spssau.com/indexs.html). PCR efficiency was estimated from the data obtained from the exponential phase of each individual amplification plot. The expression level of each gene of interest (GOI) is presented as 2^−∆∆Ct^; where ∆∆Ct = ∆Ct_GOI_-∆Ct_Contorl_; ∆Ct_GOI_ = Ct_GOI_-Ct_Actin_; ∆Ct_Contorl_ = Ct_Control_-Ct_Actin_.

## Results

### Genome-Wide Identification of *StTGA* Genes

In total, 42 *StTGA* genes were identified in *S. tuberosum*, and they were named *StTGA01–StTGA42*. This study revealed that the largest number of amino acids is 488 in the *StTGA* gene family, and the relative molecular weight of these genes is between 17,290.85 and 54,371.83 Da. In addition, we predicted the pIs of StTGA protein. The results showed that the pI of StTGA17 is 4.96, which is the lowest in the StTGA protein, while the pI of StTGA01 is 9.1, which is the highest. Furthermore, we speculated the StTGA may be distributed in many parts of the cell and most of StTGA proteins are located in cytoplasm and nucleus (Supplementary Table S1).

### *In Silico* Chromosomal Location, Phylogenetic Relationship, Exon–Intron Structure, and Conserved Motif Analysis

**FIGURE 1:**
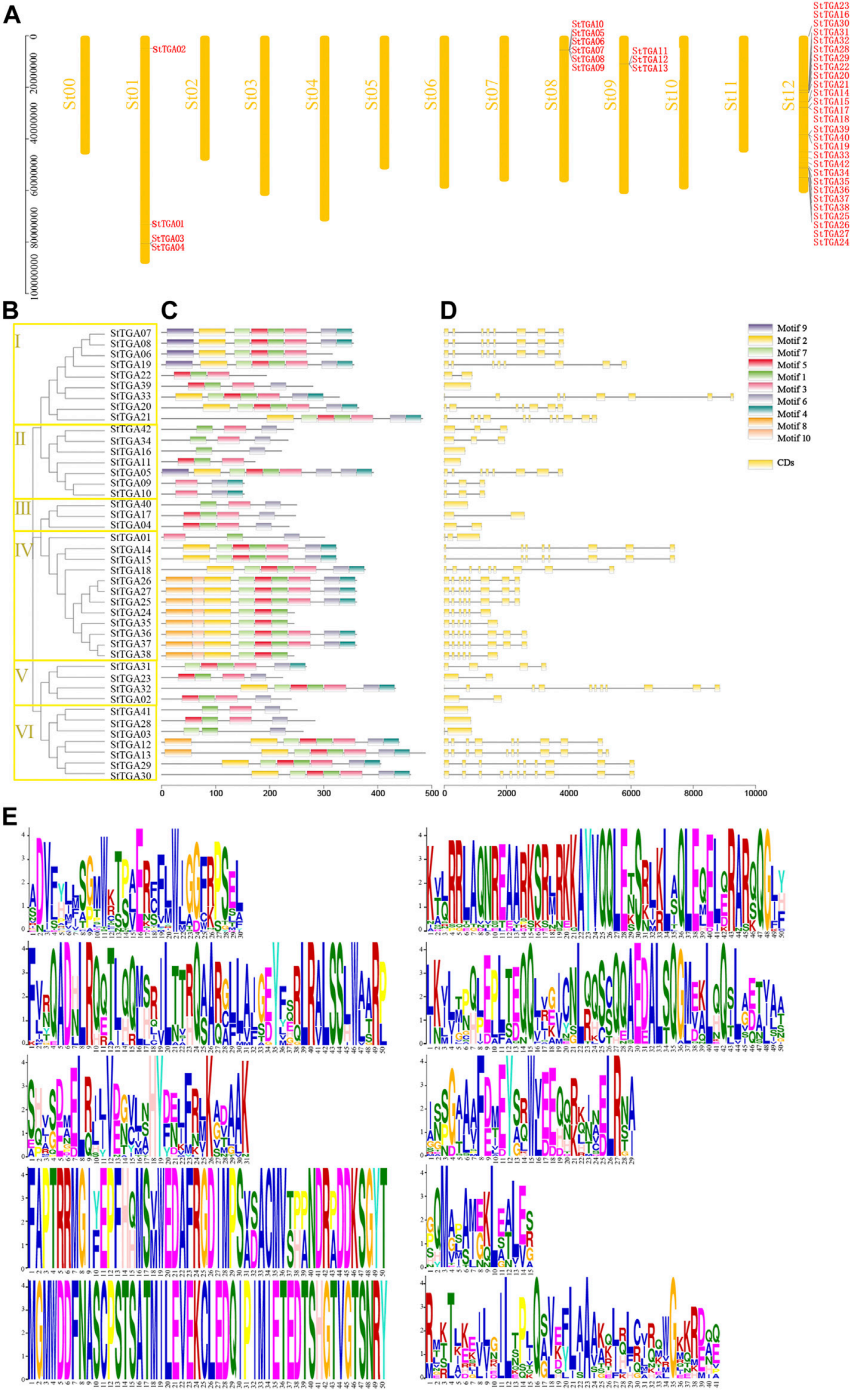
Chromosomal location, phylogenetic relationship, exon–intron structure, and conserved motif analysis of StTGA protein. **(A)** Chromosomal locations of StTGA genes. The gene names of StTGA are shown in red to the right of the chromosome. **(B)** Phylogenetic tree of potato StTGA based on its amino acid sequence. Branch lines with different colors indicated different subgroups. The proteins on the tree can be divided into six distinct subfamilies, which are indicated by different colored backgrounds. **(C)** Conserved motif of StTGA proteins analyzed by online program MEME server. Different colored boxes indicated different motifs. **(D)** Gene structure of *StTGA*. The exon–intron structure of *StTGA* genes visualized by online tool GSDS 2.0, yellow boxes indicated exons, and black lines indicated introns. **(E)** Amino acid composition of 10 conserved motifs.

Using TBtools, we drew the location map of *StTGA* and found they are distributed on four chromosomes of potato (Figure 1A). Chromosome St12 had 29 *StTGAs*, chromosome St08 contains six, St01 with four, and St09 contain three *StTGAs*. In order to study the StTGA family, we constructed the phylogenetic tree by MEGA 7 *via* the NJ method, which is shown in Figure 1B. The StTGA family was divided into six groups (designated classes I to VI) with 9, 7, 3, 12, 4, and 7 StTGAs. Then, the exon–intron structure of *StTGAs* was further analyzed. The number of exons of *StTGA* genes was between 1 and 11, of which six genes contained only one exon, and 42.86% of genes had five exons or less Figure 1D. Furthermore, 10 high conserved motifs were predicted in Figure 1C and Figure 1E. Compared to other classes, motifs 10 were exclusively found in classes IV, motifs 9 in classes I and II, and motifs 8 in classes IV and VI, separately Figure 1C.

### Cis-Acting Element Prediction Analysis of *StTGAs*

**FIGURE 2:**
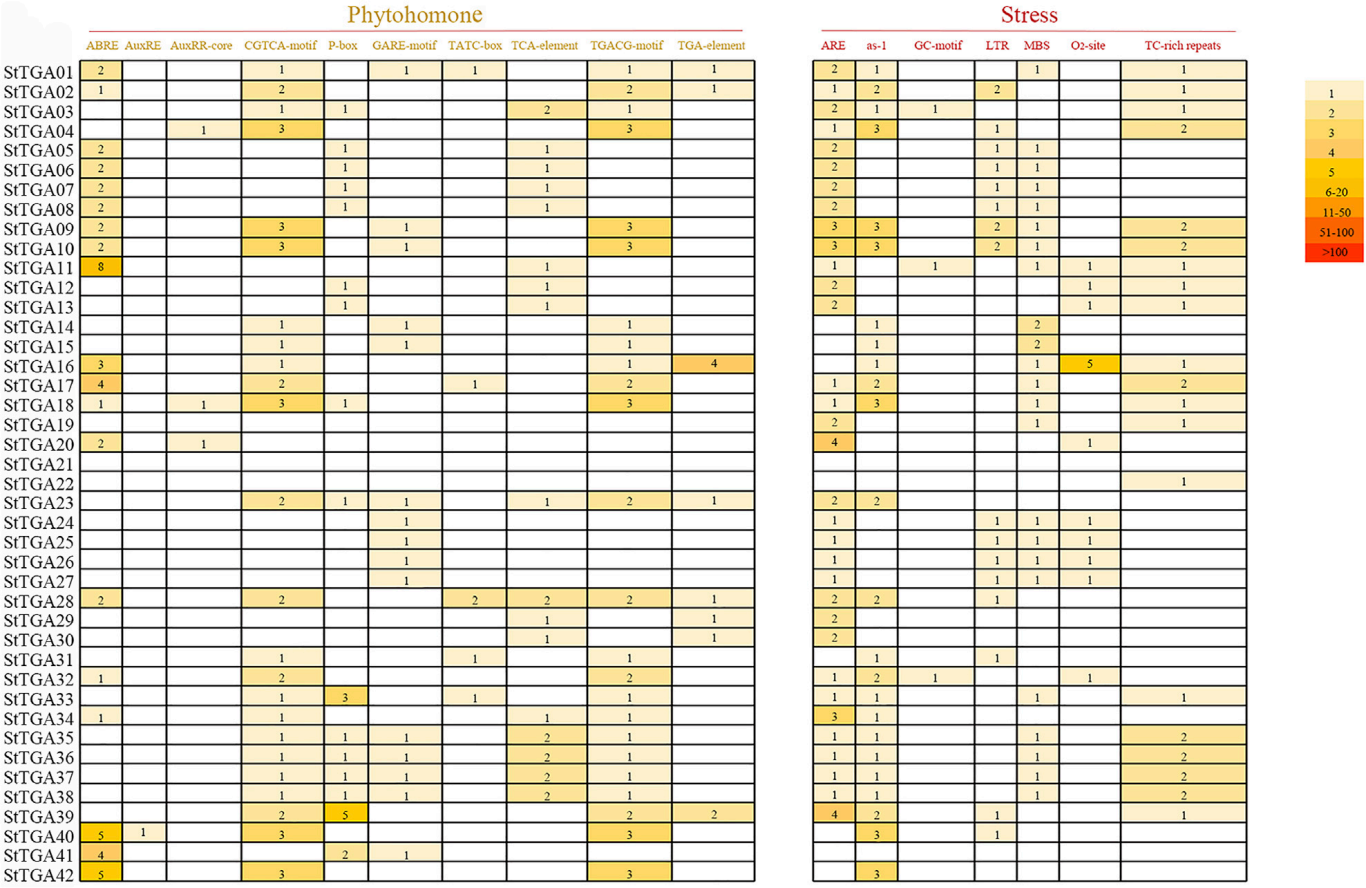
Cis-acting elements analysis in the promoter regions of *StTGA*. The promoter cis-elements were analyzed by PlantCARE, and the number of ciselements was searched in the 2,000 bp region upstream of the translation initiation site of the *StTGA* gene. Cis-elements were divided into two different types: phytohomone and stress. Expressed by different colors according to their number. The darker the color, the higher the frequency of appearance, and the number indicates the number of cis-elements.

In order to study the transcriptional regulation, the 2000 bp promoter region upstream of the *StTGA* genes was extensively analyzed by PlantCARE. The cis-elements responding to abiotic/biotic stresses and hormone treatments with the prediction score being greater than or equal to 5 were considered for further analysis (Figure 2). The results showed that the numbers and distribution patterns of the cis-elements also greatly varied among the promoters. This means that the *StTGA* genes might have different regulatory mechanisms in expression, implying their functional divergence in responses to abiotic/biotic stress and phytohormone treatments.

### Collinear Analysis of *StTGAs*

**FIGURE 3:**
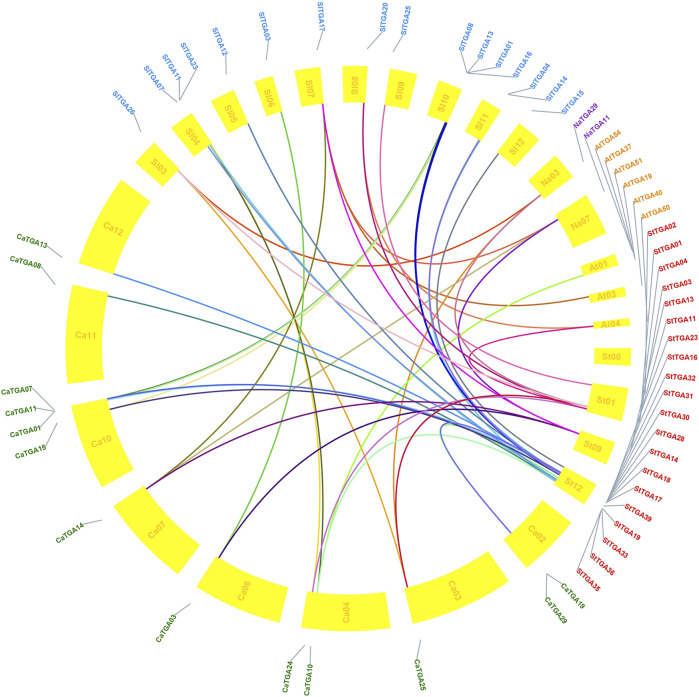
Comparative orthologous relationships of *TGA* from five species. TBtools was used to analyze the gene homology relationship between potato, tomato, pepper, Arabidopsis, and tobacco *TGA* gene families and visualize the gene homology relationship. *TGA* genes connecting five species genome are shown in colored links.

To study the evolutionary relationship of *StTGA*, we selected *S. tuberosum* as the core and identified collinearities between tomato, tobacco, Arabidopsis, pepper, and potato *TGA* genes. There are 46 pairs of one-to-one microsynteny relations (Figure 3). Some of the genes could not be assigned to any of the chromosomes. In addition, 21 orthologous gene pairs were detected between potato and tomato, 20 orthologous genes were found between potato and pepper, 10 orthologous gene pairs were identified between potato and Arabidopsis, and two orthologous gene pairs were detected between potato and tobacco. The collinear relationship among these five species is shown in Supplementary Additional File S3.

### Expression Profile of *StTGAs*

**FIGURE 4:**
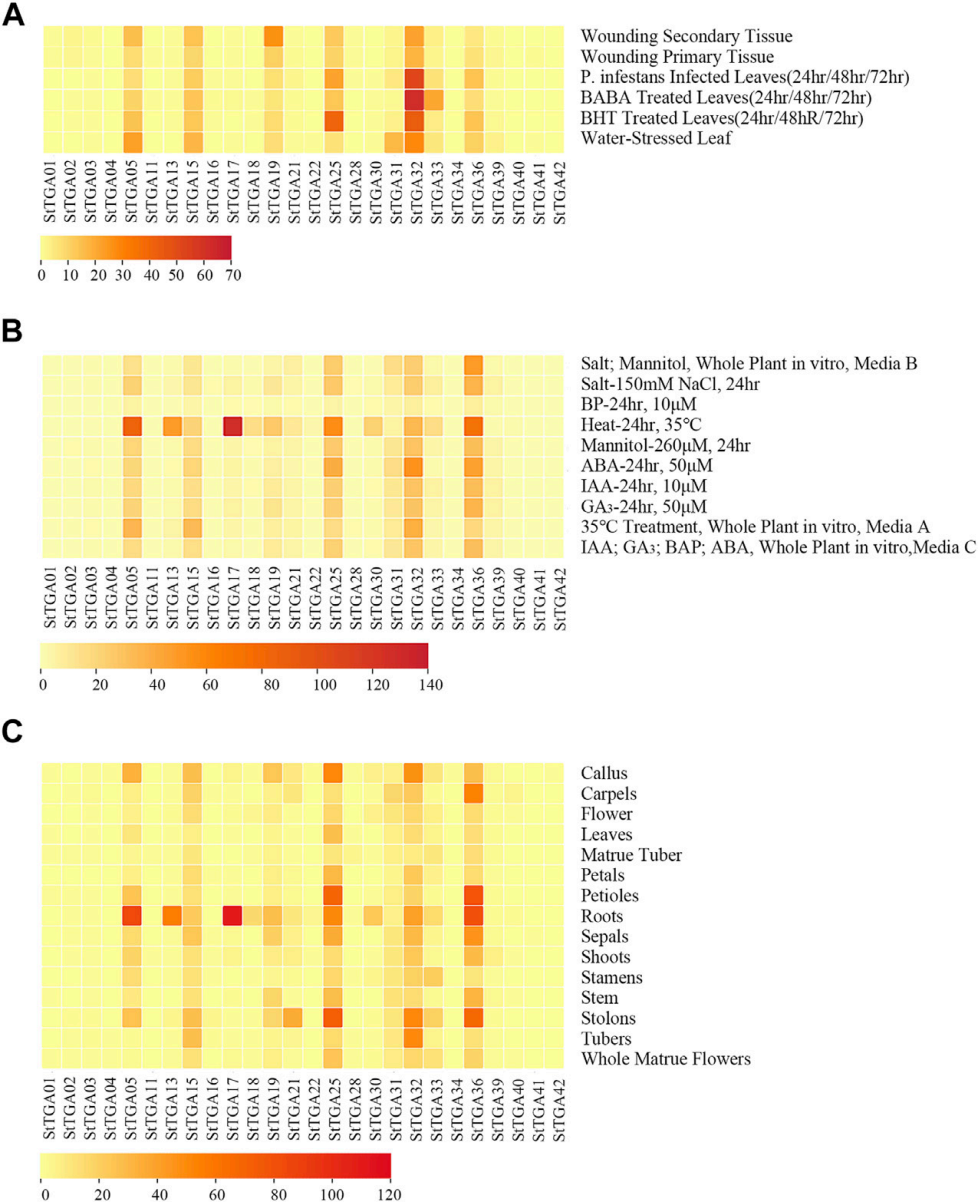
Heat map of *StTGA* genes under different stresses and hormone treatments in potato. The heat map displayed the *StTGA* expression patterns of treatment in leaves, whole plant, or in different tissues and organs. **(A)** In leaves, distance values range from 0.00 to 70.00, **(B)** in the whole plant, distance values range from 0.00 to 140.00, **(C)** in different tissues or organs indicated to the right of the heat map, distance values range from 0.00 to 120.00. The gene names are indicated at the bottom. Distance value range is depicted by the gradient of colors ranging from light yellow (lowest distance value indicating high similarity between genomes) to red (highest distance value indicating low similarity between genomes). Stress treatment in leaves is indicated to the right of the heat map.

**FIGURE 5:**
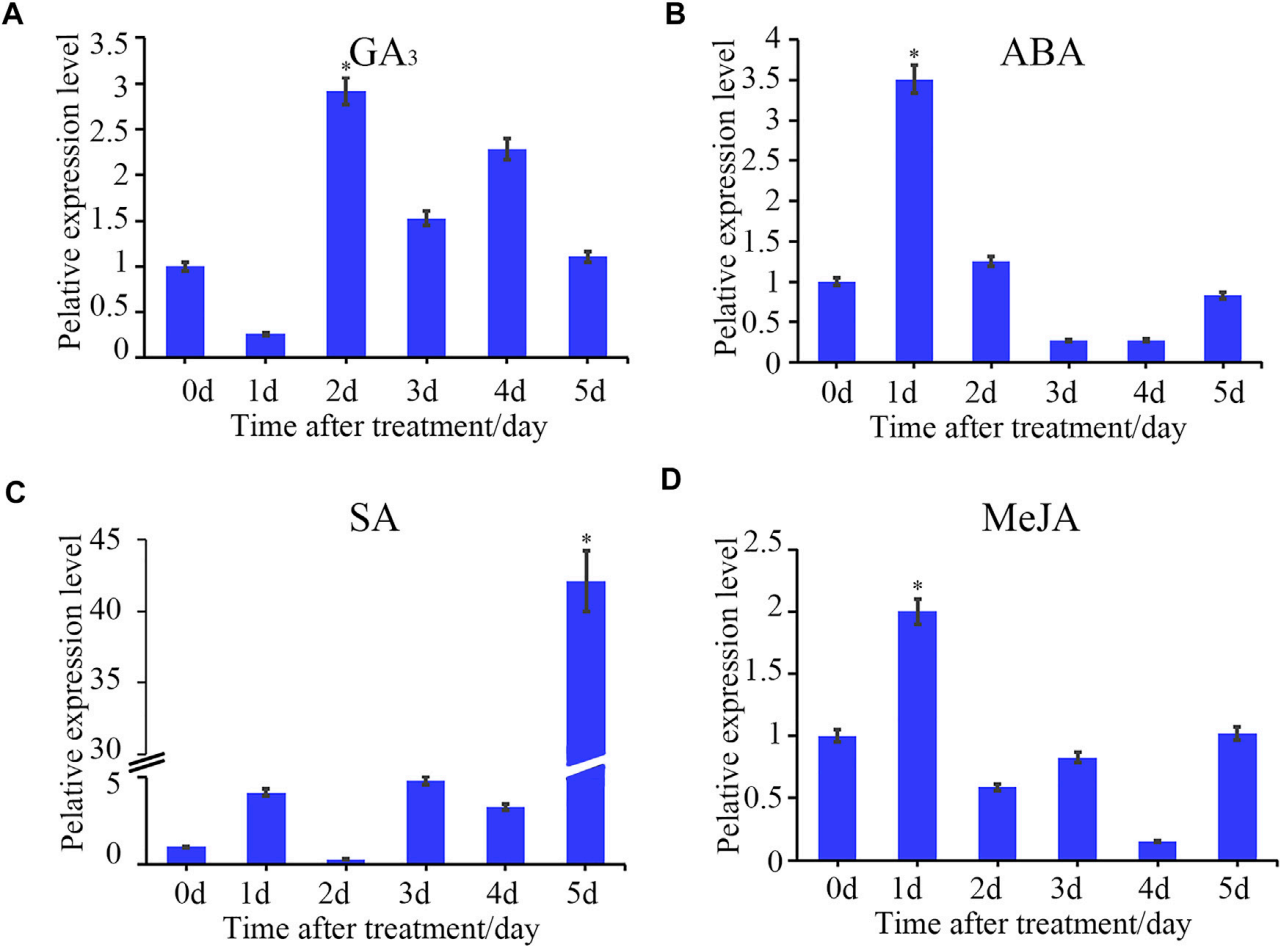
Time-course gene expression pattern of *StTGA39* induced by exogenous phytohormones. The relative expression level of *StTGA39* at different time points in potato inoculated with plant hormones **(A)** GA_3_, **(B)** ABA, **(C)** SA and **(D)** MeJA was analyzed by qRT-PCR. mRNA levels were normalized to actin (Student’s *t*-test, *n* = 3 independent experiments, data shown are mean ± standard deviation).

We have retrieved the expression data of StTGA *via* the RNA-Seq Expression Browser and the heat map of gene FPKM value constructed using the TBtools software. We found that 11 of the StTGAs (StTGA 01, StTGA 02, StTGA03, StTGA 04, StTGA 11, StTGA 16, StTGA 23, StTGA 28, StTGA 34, StTGA 41, and StTGA42) were not expressed in almost all organs. On the contrary, StTGA05, StTGA15, StTGA25, StTGA32, and StTGA36 played roles in different developmental stages and tissues (Figure 4). For verification of RNA-seq data, the potato seedlings were treated with the exogenous hormones ABA, GA_3_, SA, and MeJA, and the results showed the inducible upregulated expression of the *StTGA39* gene (Figure 5). By comparison, the effect of SA was relatively strong, while that of the MeJA, ABA, and GA_3_ was light for inducing the upregulation.

### Co-Expression Network Construction

**FIGURE 6:**
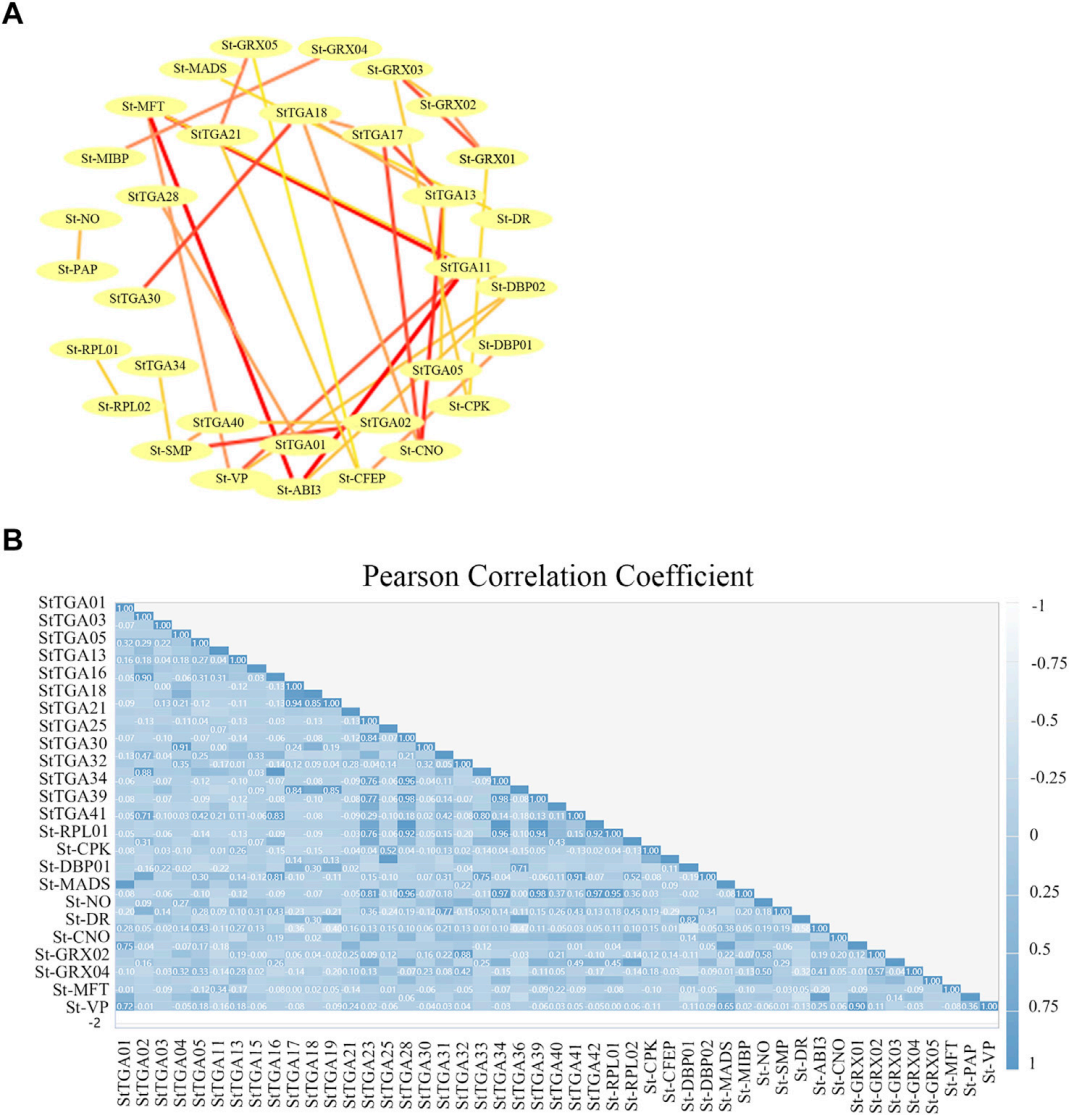
Construction of the StTGA co-expression network. **(A)** Co-expression network analyses are performed to characterize the 36 differentially expressed StTGA. In the network, nodes represented RNAs, while lines represented co-expression and prediction relationship. **(B)** PCC of all genes in each gene show in this figure. Pearson correlation matrix between all StTGAs. (positive correlations = dark blue with value, negative correlations = light blue, non-significant correlations = white).

To determine the relationship of StTGA proteins and their interactors further, we performed a gene co-expression network analysis based on the STRING database. Twenty-four other potato proteins, such as 60S ribosomal protein L18 (RPLs), DNA-binding proteins (DBPs), and Glutaredoxin (GRXs), were identified Figure 6, which exhibited a co-expression relationship with StTGA (Figure 6A). The co-expression network comprised 36 pairs, and each pair of nodes was directly or indirectly connected to each component (Figure 6A). StTGA and StTGA members, StTGA and other potato proteins presented co-expression relationships, albeit with different weight values (Figure 6B). In particular, StTGA01 (StTGA28), StTGA02 (StTGA40), StTGA05 (StTGA13), etc., presented co-expression relationships (Figure 6A).

### Prediction of StTGA Structure

**FIGURE 7:**
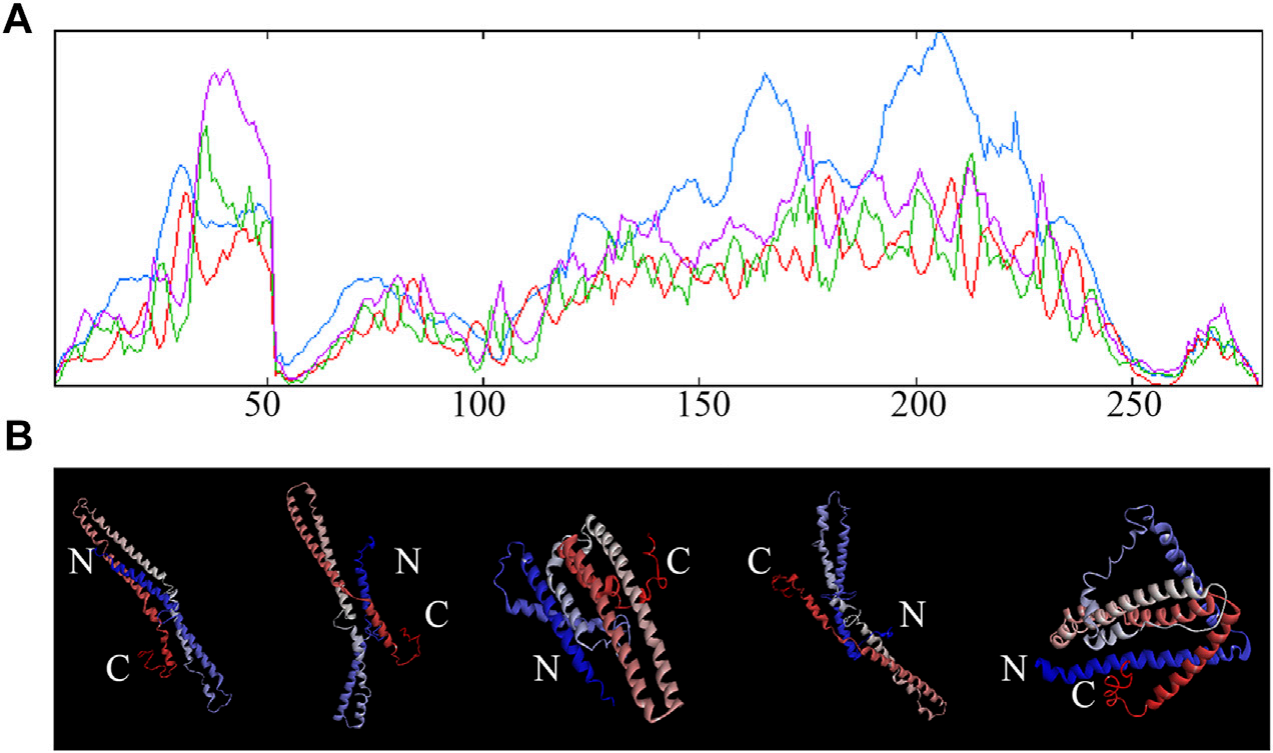
Secondary and tertiary structure of potato StTGA39 protein. **(A)** Secondary structure of StTGA39 was predicted by SOPMA in potato. Blue lines represent alpha-helix, purple lines represent random coil, red lines represent extended strand, and green lines represent *β*-turn. **(B)** Three-dimensional structure of StTGA39 was built with the I-TASSER web server.

The secondary structure of StTGA39 was predicted by SOPMA. It was found that the a-helix of StTGA protein consists of 188 amino acids, accounting for 67.14%; the random coil of StTGA protein consists of 78 amino acids, accounting for 27.86%; the extended strand of StTGA protein consists of nine amino acids, accounting for 3.21%; and the β-turn of StTGA protein consists of five amino acids, accounting for 1.79% (Figure 7A). A three-dimensional structure of StTGA39 was built with the I-TASSER web server. Five possible models of StTGA protein were predicted on the 3D structure, according to the website modeling with Discovery Studio 4.5 Client. The conservative domains of these models were basically the same (Figure 7B).

### Effect of *R. solanacearum* Infection on Potato

**FIGURE 8:**
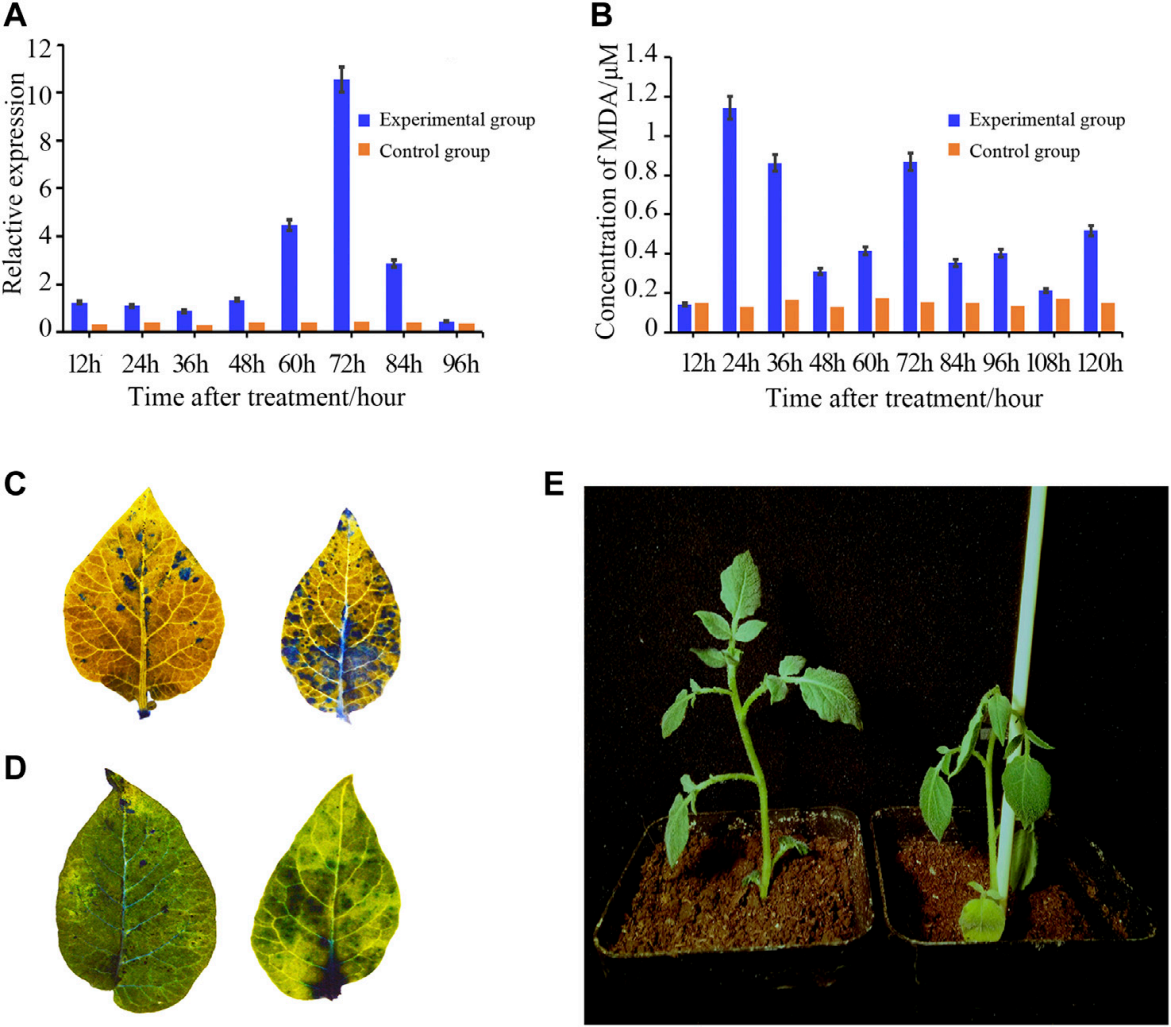
Upregulated expression pattern of *StTGA39* and concentration of MDA during R. solanacearum infection on potato. **(A)** Expression pattern of *StTGA39* was induced by R. solanacearum. **(B)** Levels of MDA in potato plants were assessed at 12, 24, 36, 48, 60, 72, 84, 96, 108, and 120 hpi inoculated with R. solanacearum. **(C)** Phenotype of two-week-old potato leaves after 72 hpi with flg22 treatment was compared with WT. **(D)** Cell death in potato was detected by histochemical staining with trypan blue staining. **(E)** Photograph showed the phenotype of the WT and was taken 3 days after R. solanacearum infection. mRNA levels were normalized to actin (Student’s t-test, *n* = 3 independent experiments, data shown are mean ± standard deviation).

In order to confirm whether *StTGA39* was induced by *R. solanacearum*, the induced expression of *StTGA39* mRNA in potato seedlings inoculated with *R. solanacearum* was compared and analyzed by the qRT-PCR method (Figure 8A). We found that the expression level of *StTGA39* was low when plants were inoculated with water. In comparison with the control group, the expression increased at 60 hpi and reached the highest level at 72 hpi after inoculating with the highly pathogenic strain PO41. That is to say, the expression of the *StTGA39* gene was up-regulated when stressed by *R. solanacearum* and may play an important role in potato resistance to bacterial wilt. Similar results have been confirmed by other family members of *StTGA* (Supplementary Additional File S4).

In order to determine the defense response, various histochemical analyses were carried out on potato leaves (Figure 8). Under the stress of *R. solanacearum* solution of 10^8^ cfu/L, the effect of MDA content in potato seedling leaves is shown in Figure 8B. It could be seen from Figure 8B that with the prolongation of infection time of *R. solanacearum*, the change of MDA content in potato seedling leaves showed a trend, which was upregulated once at 24 h and then decreased at intervals of 24 h. Callose strengthens the plant cell walls and prevents cells from being invaded by pathogens. Aniline staining analysis and trypan blue analysis showed that the induction of callose deposition and the HR-like cell death was significantly increased in the treated potato leaves (Figure 8C and Figure 8D).

### Y1H Assay

**FIGURE 9:**
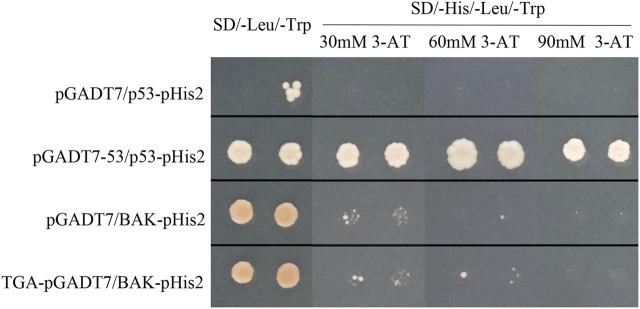
Y1H showing the association of StTGA39 with the promoter of the StBAK1-20 gene. Y1H assay for determination of StTGA39-StABAK1-20 interaction. Growth of yeast cells transformed with the effector vector and the reporter vector on SD (-His, -Leu, -Trp) supplemented different concentrations of 3-AT. The positive control is pGADT7-53/p53-pHis2 vector and negative control is pGADT7/p53-pHis2 vector.

*BAK1* is a well-established receptor for defense-related genes in plants. To identify if StTGA1 could interact directly with *StBAK1*, Y1H analysis was used (Figure 9). The yeast expression vector TGA-pGADT7 and BAK-pHis2 were constructed. The results of Y1H showed that all the Y1H gold strains co-transformed with pHIS2 vectors and clones grew on SD (-Leu, -Trp) medium, indicating that the co-transformation was successful. The growth was different at different concentrations of 3-AT-deficient SD (-His, -Leu, -Trp) medium. On SD (-His, -Leu, -Trp) medium, the growth inhibition of the pGADT7/BAK-pHis2 self-activated group deepened with the increase of 3-AT concentration. The growth condition of the TGA-pGADT7/BAK-pHis2 group was slightly better than that of the control group, although the experimental group could not grow on SD (-His, -Leu, -Trp; 90 mM) (Figure 9). The results showed that StTGA1 might bind to *StBAK1*.

## Discussion

TGA TFs have been reported to function in various biological processes in plants (Jiang et al., 2021; Martins et al., 2022) and members of the TGA TFs play crucial roles in response to microbial pathogens in plants, such as Arabidopsis, soybean, rice, and tobacco (Pontier et al., 2002; Van et al., 2011; Moon et al., 2018; Ullah et al., 2019). There is a lack of reports on the potential link between TGA TFs and resistance to bacterial wilt caused by R. solanacearum in potato. In the present study, 42 StTG*A* genes were identified based upon the entire genome sequences of the potato, and the total number of TGA in potato was slightly expanded compared to that in tomato, tobacco, and pepper identified in this study but lower than that in Arabidopsis (Supplementary Additional File S1), which was different from the earlier reports (Ullah et al., 2019). This was mainly due to the continuous updates in the database and the different approaches used by authors. In addition, the phylogenetic analysis and collinearity was carried out. The results indicated that the StTGA family is conserved in the evolutionary history with the TGA TFs orthologs of other plants. This result corresponds well with previous observations in many plant species (Moon et al., 2018; Ullah et al., 2019; Jiang et al., 2021).

In order to investigate the expression patterns in response to different abiotic and biotic stresses, 10 members were selected for further study. The expression heat map demonstrated that the TGA TFs may have played significant and complex roles in potato (Figure 4), which was reflected in at least two aspects. First, the RNA-seq data-based heat map showed that the expression of most of the StTGAs was significantly different under abiotic/biotic stresses, which was consistent with previous studies (Jiang et al., 2021). Second, the same gene showed different or even opposite expression patterns under different stresses or in different tissues. For instance, the StTGA17 gene was upregulated in leaves under heat stress but downregulated under salt stress (Figure 4B). These results illustrate that these genes are widely involved in the response to various stresses and participate in a complex cross-regulatory network and signaling pathways (Duan et al., 2022).

To uncover the possible signal-associated functions of *StTGAs* in response to hormone stress, we conducted qRT-PCR to analyze their relative expressions under SA, MeJA, ABA, and GA_3_ treatments (Figure 5). This induction may be related to the upstream cis-element elements in the promoters of the genes. According to the analysis of cis-acting elements (Figure 2) and the results of qRT-PCR (Figure 5), we speculated StTGA could be induced by multiple phytohormones and might play a crucial role in multiple hormone signal pathways. Consistent with our results, some cis-elements play an important role in responses to ABA (Baker et al., 1994; Busk and Pagés, 1998; Li J. et al., 2019). There is an interaction between NPR1 and TGA TFs, which eventually leads to activation of SA-dependent response (Rahman et al., 2012). Studies have shown that a *R. solanacearum* effector targets TGA TFs to subvert SA signaling (Qi et al., 2022). Furthermore, the JA signal pathway is related to the function of *Tripterygium wilfordii hook*. F. *TGA1* (*TwTGA1*) (Han et al., 2020). All of these research studies further support our idea that *TGA* may play a key role in the regulation of these hormone signal pathways.

An important goal in this study was to speculate the function of the *StTGA* gene in the response mechanism of potato to *R. solanacearum.* In potato, the invasion of *R. solanacearum* could cause up-regulation of *StTGA* gene expression according to Figure 8A. The result showed that *StTGA* was involved in resisting the invasion of *R. solanacearum*. Although the detailed mechanism needs further study, to prove this view, our study showed some other data related to resistance. Plants could resolve pathogens such as bacteria by causing ROS burst (Li et al., 2016; Ferreira et al., 2017). Evidence revealed that TGA class II plays an important role in the tolerance response to control ROS levels in Arabidopsis (Ariel et al., 2021). In addition, ROS induces gene expression and stress response and has regulatory roles in a wide range of important plant biological processes (Xia et al., 2010). Consistent with this possibility, R. solanacearum infection can result in enhanced production of ROS and lead to ROS-related oxidative damage (Ferreira et al., 2017). The results of DAB staining confirmed there is a mechanism in S. tuberosum to resist this adverse effect (Figure 8D). Furthermore, MDA can indicate the degree of membrane per-oxidation and further support the results of DAB staining (Figure 8B). The abovementioned views further support StTGA TFs are crucial regulatory factors in potato resistance to bacterial wilt, and our further data on identification of the target gene of this TF seem to support this view.

TGA TFs recognize the TGACG-motif (TGA-binding site) within the promoters of their target genes (Hou et al., 2019). To detect whether StTGA39 binds to the TGACG-motif present in the *StBAK1-20* promoter (Supplementary Additional File S5) in yeast, the Y1H assays were performed, and the result suggested that StTGA39 specifically binds to the TGACG-motif in the promoter of the *BAK1* gene which plays an essential role in regulated plant immunity (Beg et al., 2013). The results showed that the *StBAK1-20* gene involved in the PAMP-triggered immunity (PTI) signal pathway (Beg et al., 2013) and interacted with StTGA in our materials. The abovementioned results indicated that the StTGA can function by combining with the promoter region of *StBAK1-20* (Supplementary Additional File S6). To our knowledge, there are few reports on the TGA-promoted transcription of BAKs despite recent reports that TGAs directly activate respiratory burst oxidase homolog D (RBOHD) and pathogenesis-related protein 1 (PR1) expression in Arabidopsis (Qi et al., 2022; Shimizu et al., 2022).

## Conclusion

To sum up, we conducted a genome-wide analysis of the *StTGA* gene family in potato and primarily explored the role of them. A total of 42 *StTGA* genes were identified from the potato genome. The structure diversity, chromosomal distribution, and evolutionary history of *StTGA* were comprehensively analyzed. These results extended the understanding on the abundance and diversity of *StTGA* genes in this important crop, which may serve as a fundamental resource for the molecular breeding of potato. However, the detailed molecular mechanism needs to be further studied. Taken together, this study lays the foundation for further investigation of *StTGA* in potato.

## Data Availability

The original contributions presented in the study are included in the article/supplementary materials; further inquiries can be directed to the corresponding author.
