# Supplementary material for: A Genome-Wide Analysis of StTGA Genes Reveals the Critical Role in Enhanced Bacterial Wilt Tolerance in Potato During Ralstonia solanacearum Infection
Source: Front Genet. 2022 Jul 26;13:894844. doi: 10.3389/fgene.2022.894844 (PMC9360622; doi:10.3389/fgene.2022.894844)
Supplement: Supplementary file 1 [file DataSheet1.zip › Supplementary Additional File S5. sequence of StBAK1-20 gene showing the StTGA39 TF binding site in the promoter region.pdf]

>sequence of *StBAK1-20* gene showing the StTGA39 TF binding site in the promoter region

GTAACCGAACTGGCGAAAGAAGCTATATTATACTTGGAGAGAGGGACGTTATTTCATAGTAGATATTC**CGTCA**CTT

StTGA39 TF binding site

TATCCTATTTGAGTTATGCAACATTATGCTATGTTTTTTTTTTTTTTGAGAAGGTAACATTTGTGTATATTCATTG

binding site

ACCCAGTGCATGGGTTGCAGTGTAAACCATATTTACAGCTTGTCAAAAAAGTAAGAAAACTGAATCTGAATCCTAA
CAGGAACCTAGGATATCTATAATGGATTCAGTGTCCTCTAAATACATCTGCTTGCACCAGAAGCAAAAAAGCCTCA
GACAATTTAGTTTGATCTTTTGAAGATCACAGCTCTTGGTCTCAAAACATCTAGCATTTCTTTCTTTCCAAACAGT
CCACCAAATACAGGCTAGGACAATCCTCCATCTATCTCTATCTGCAGCTCCCACATTACGCTATGGTTTTGGTGAT
ATGAATAATTTTCATTTTACTTTATTAACCATGTATTCATTTTCTGAGGGTACATTTGCTCAGAATTAATTGATAA
GATTGTTCTTTAATACCTTATATAGTGATGTTAACAGATATTTGATTTGCTCATTCTACTGCCCTATGTAATGTTC
TCATTGCTACCCTTTTGGACCAGTCCTCCAGTGGAAGTTGGTGCAAATAACCTATCCTGCTTCCATTATATTGATG
TTGTAGTTTTTGTGGTGGTAGAATCCTCTGAACATAAAATCCTCTCAACATGAAGTATGTTAACTTGGCTCCTTAT
TCCCTCAAGCTTCTTTTCTTCTATTTTTTAAGAAGCGATTAATGGTGTTTTGAACTATTTTCGCGCAGATTGACAA
TGAAAGTTTAATTTAGTCAAATAGAACCAATTATTCAATTACAGAGTTATCTACACGAGGTAAAAAATAAACCGCC
TTCCAGGAAAATTGAACCCTGAATCTTGAAATCCAGAGATCTCTAACTTGCCACTTGATCTAGGTGTTGTTTACTA
CCACCTGATTATTTGCTTGCCAAATTCTGAAGTTGTGTGCTAAAATCACGATACTCTCTGTTTGGGTGGGGAACTT
CCTTAGTTACTAGTCTACACTATAGTTATACCTTACCTATTGCTTTTAGGGGTCGTTTGGTAGGAGTTGTTAGAGA
AAATAGTACTGGTATCAGCTTTGGTATTATTTAATCCCTTGTTTCGTAGTGTTCTCAACCTATGTATAACTAATAC
ACCCTATTCAATACTATTCTTATACATAGTAAATCATGACATTAGCAATACCAGTACTATTCATATTCTAATACAC
CCAATTTATATAATACAACAAACCAAATAGTCGACAAAAAATAGTCTAGCATAACTAATCTCATTATTACTAATAC

TATA box

ACCCTATTCAGTTCTATTATTAATTATACCCCTACCAAACGACCCTTTAGTACATCTGTTCTTTTTTTCATGTTTA
CCTAAGAGTGTAACTTGTGGAAGTAATTTACACTTCAAATGTGCAACATAATTATGCACATGTTAGTTGGCATGTC
TCATCTTAAGCGGCATGGTGGGCAAATTCTCAGAATAACTTGCTAATGATCTTTTTTTTTAATTTCAGGGAACTTT

5' UTR

ATAGTAATAACATAAGCGGAAGAATTCCAAATGAACTGGGGAACTTGACAGAGTTGGTTAGTTTGGATCTTTACCT
GAACAACTTAAATGGTCCTATCCCTCCCTCATTGGGCAAGCTTCAGAAACTACGCTTCCTGAGGCTCAATAATAAC
AGTTTGAATGAAGGTATTCCCGTGTCTCTAACCACCATTGTTGCACTTCAAGTACTTGATCTCTCAAACAACCATT
TGACAGGACCAGTTCCAGTCAACGGTTCCTTTTCACTTTTTACTCCTATAAGTTTTGCTAATAATCAGTTGGAAGT
TCCTCCAGTTTCTCCACCTCCTCCCCTTCCTCCTACGCCCTCATCGTCATCTTCAGTGGGCAACAGCGCAACTGGA
GCTATCGCTGGAGGAGTTGCTGCAGGCGCTGCCCTTCTATTTGCAGCTCCTGCAATTTTTATTGCTTGGTGGCGTC
GGAGGAAACCGCAAGACCACTTCTTTGATGTTCCTGCTGAGGAGGATCCAGAAGTTCATCTGGGACAACTCAAAAG
GTTTTCCTTGCGTGAACTACAAGTTGCGTCGGATAATTTTAGCAACAGAAATATACTCGGTAGAGGTGGATTTGGT
AAGGTTTATAAAGGCCGGTTAGCTGATGGCTCTTTAGTTGCAGTGAAAAGACTAAAAGAGGAACGTACTCAAGGTG
GAGAGTTACAGTTTCAGACAGAAGTAGAAATGATCAGCATGGCTGTACACCGAAACCTACTTCGTTTACGGGGATT
TTGCATGACACCCACTGAGCGGGTGCTTGTTTATCCGTACATGGAGAATGGAAGTGTTGCATCACGTTTAAGAGAG
AGGCCTGAATCAGAGCCCCCACTTGACTGGCCAAAAAGGAAGCGTATTGCACTTGGATCTGCAAGAGGCCTTGCTT
ACTTGCATGATCATTGTGATCCTAAAATTATTCATCGTGACGTCAAAGCCGCAAATATCTTGTTGGATGAGGAGTT
TGAAGCAGTTGTTGGGGATTTTGGGTTAGCTAAACTCATGGACTACAAGGATACTCATGTTACCACTGCTGTACGT
GGTACAATTGGGCATATTGCCCCTGAATATTTATCTACTGGTAAATCTTCTGAGAAAACTGATGTGTTTGGCTATG
GGGTTATGCTTCTAGAGCTCATAACTGGGCAAAGGGCTTTTGATCTTGCTCGACTTGCGAATGATGATGATGTCAT
GCTGCTAGATTGGGTGAAGGGACTCCTGAAGGACAAGAAATATGAAACATTAGTTGATGCAGATCTTCAAGGTAAT
TACAATGAAGAAGAAGTGGAACAGCTTATTCAGGTAGCTCTACTTTGCACGCAGAGTACGCCTACGGAACGTCCAA
AGATGTCAGAAGTTGTAAGAATGCTTGAAGGTGATGGCCTTGCTGAGAGGTGGGAGGAATGGCAAAAGGAGGAGAT
GTTCCGGCAAGATTTCAACCATGTCCACCACCACCATACTGATTGGATAATAGCTGACTCCACTTCAAATATCCGA
CCGGATGAGTTGTCAGGGCCAAGATGA
