# Supplementary material for: A Genome-Wide Analysis of StTGA Genes Reveals the Critical Role in Enhanced Bacterial Wilt Tolerance in Potato During Ralstonia solanacearum Infection
Source: Front Genet. 2022 Jul 26;13:894844. doi: 10.3389/fgene.2022.894844 (PMC9360622; doi:10.3389/fgene.2022.894844)
Supplement: Supplementary file 1 [file DataSheet1.zip › Supplementary Additional File S6. BAK1 promoter.pdf]

>StBAK1-20

GTCAATTTTGTGTGCATAAAAATTTGCTCATGTAGCTTCTCGACTTATTAGAAGTGTACTGCCCCATCAAGTTGATTTTAT

GAATCATCCACACCCTAATTTAAAATATCCAACCTTAATTTTTCTAGGAGGAACCAACGCATTTCTGTTCATGTACTTGGA

TATTCTTTTGATTGCAAAGATGCAAATTTGACTAGTTATCGGAAAATGAAAGAAAAAACCAGTTTGGCTGGTGATTCTCCT

TCTAGTCCCAATTTACCTGATCATTTACTAAATAAAAAAGCTTTTTTTGTATTCAAGAATTAAAATTACAAAAATAGTATT

GCTTGATATTTTCTGAAGGCCACTCCACTTTATGACATTAACAACTATTATTCTATTTTACTTTTTTTCAAGTTAAATTTT

ATATGACAATGCTGGTCAAATTCTTTTTTTGTAACCGAACTGGCGAAAGAAGCTATATTATACTTGGAGAGAGGGACGTTA

TTTCATAGTAGATATTCCGTCACTTTATCCTATTTGAGTTATGCAACATTATGCTATGTTTTTTTTTTTTTGAGAAGGTA

TGA binding site

ACATTTGTGTATATTCATTGACCCAGTGCATGGGTTGCAGTGTAAACCATATTTACAGCTTGTCAAAAAAGTAAGAAAACT

GAATCTGAATCCTAACAGGAACCTAGGATATCTATAATGGATTCAGTGTCCTCTAAATACATCTGCTTGCACCAGAAGCAA

AAAAGCCTCAGACAATTTAGTTTGATCTTTTGAAGATCACAGCTCTTGGTCTCAAAACATCTAGCATTTCTTTCTTTCCAA

ACAGTCCACCAAATACAGGCTAGGACAATCCTCCATCTATCTCTATCTGCAGCTCCCACATTACGCTATGGTTTTGGTGAT

ATGAATAATTTTCATTTTACTTTATTAACCATGTATTCATTTTCTGAGGGTACATTTGCTCAGAATTAATTGATAAGATTG

TTCTTTAATACCTTATATAGTGATGTTAACAGATATTTGATTTGCTCATTCTACTGCCCTATGTAATGTTCTCATTGCTAC

CCTTTTGGACCAGTCCTCCAGTGGAAGTTGGTGCAAATAACCTATCCTGCTTCCATTATATTGATGTTGTAGTTTTTGTGG

TGGTAGAATCCTCTGAACATAAAATCCTCTCAACATGAAGTATGTTAACTTGGCTCCTTATTCCCTCAAGCTTCTTTTCTT

CTATTTTTTAAGAAGCGATTAATGGTGTTTTGAACTATTTTCGCGCAGATTGACAATGAAAGTTTAATTTAGTCAAATAGA

ACCAATTATTCAATTACAGAGTTATCTACACGAGGTAAAAAATAAACCGCCTTCCAGGAAAATTGAACCCTGAATCTTGAA

ATCCAGAGATCTCTAACTTGCCACTTGATCTAGGTGTTGTTTACTACCACCTGATTATTTGCTTGCCAAATTCTGAAGTTG

TGTGCTAAAATCACGATACTCTCTGTTTGGGTGGGGAACTTCCTTAGTTACTAGTCTACACTATAGTTATACCTTACCTAT

TGCTTTTAGGGGTCGTTTGGTAGGAGTTGTTAGAGAAAATAGTACTGGTATCAGCTTTGGTATTATTTAATCCCTTGTTTC

GTAGTGTTCTCAACCTATGTATAACTAATACACCCTATTCAATACTATTCTTATACATAGTAAATCATGACATTAGCAATA

CCAGTACTATTCATATTCTAATACACCCAATTTATATAATACAACAAACCAAATAGTCGACAAAAAATAGTCTAGCATAAC

TAATCTCATTATTACTAATACACCCTATTCAGTTCTATTATTAATTATACCCCTACCAAACGACCCTTTAGTACATCTGTT

CTTTTTTTCATGTTTACCTAAGAGTGTAACTTGTGGAAGTAATTTACACTTCAAATGTGCAACATAATTATGC

ACATGTTAGTTGGCATGTCTCATCTTAAGCGGCATGGTGGGCAAATTCTCAGAATAACTTGCTA

5’ UTR

ATGATCTTTTTTTTTAATTTCAGGGAACTTTATAGTAATAACATAAGCGGAAGAATTCCAAATGAACTGGGGAACTTGACA

initial codon “ATG”

GAGTTGGTTAGTTTGGATCTTTACCTGAACAACTTAAATGGTCCTATCCCTCCCTCATTGGGCAAGCTTCAGAAACTACGC

TTCCTGAGGCTCAATAATAACAGTTTGAATGAAGGTATTCCCGTGTCTCTAACCACCATTGTTGCACTTCAAGTACTTGAT

CTCTCAAACAACCATTTGACAGGACCAGTTCCAGTCAACGGTTCCTTTTCACTTTTTACTCCTATAAGTTTTGCTAATAAT

CAGTTGGAAGTTCCTCCAGTTTCTCCACCTCCTCCCCTTCCTCCTACGCCCTCATCGTCATCTTCAGTGGGCAACAGCGCA

ACTGGAGCTATCGCTGGAGGAGTTGCTGCAGGCGCTGCCCTTCTATTTGCAGCTCCTGCAATTTTTATTGCTTGGTGGCGT

CGGAGGAAACCGCAAGACCACTTCTTTGATGTTCCTGCTGAGGAGGATCCAGAAGTTCATCTGGGACAACTCAAAAGGTTT

TCCTTGCGTGAACTACAAGTTGCGTCGGATAATTTTAGCAACAGAAATATACTCGGTAGAGGTGGATTTGGTAAGGTTTAT

AAAGGCCGGTTAGCTGATGGCTCTTTAGTTGCAGTGAAAAGACTAAAAGAGGAACGTACTCAAGGTGGAGAGTTACAGTTT

CAGACAGAAGTAGAAATGATCAGCATGGCTGTACACCGAAACCTACTTCGTTTACGGGGATTTTGCATGACACCCACTGAG

CGGGTGCTTGTTTATCCGTACATGGAGAATGGAAGTGTTGCATCACGTTTAAGAGAGAGGCCTGAATCAGAGCCCCCACTT

GACTGGCCAAAAAGGAAGCGTATTGCACTTGGATCTGCAAGAGGCCTTGCTTACTTGCATGATCATTGTGATCCTAAAATT

ATTCATCGTGACGTCAAAGCCGCAAATATCTTGTTGGATGAGGAGTTTGAAGCAGTTGTTGGGGATTTTGGGTTAGCTAAA

CTCATGGACTACAAGGATACTCATGTTACCACTGCTGTACGTGGTACAATTGGGCATATTGCCCCTGAATATTTATCTACT

GGTAAATCTTCTGAGAAAACTGATGTGTTTGGCTATGGGGTTATGCTTCTAGAGCTCATAACTGGGCAAAGGGCTTTTGAT

CTTGCTCGACTTGCGAATGATGATGATGTCATGCTGCTAGATTGGGTGAAGGGACTCCTGAAGGACAAGAAATATGAAACA
TTAGTTGATGCAGATCTTCAAGGTAATTACAATGAAGAAGAAGTGGAACAGCTTATTCAGGTAGCTCTACTTTGCACGCAG
AGTACGCCTACGGAACGTCCAAAGATGTCAGAAGTTGTAAGAATGCTTGAAGGTGATGGCCTTGCTGAGAGGTGGGAGGAA
TGGCAAAAGGAGGAGATGTTCCGGCAAGATTTCAACCATGTCCACCACCACCATACTGATTGGATAATAGCTGACTCCACT
TCAAATATCCGACCGGATGAGTTGTCAGGGCCAAGATGA

TCTTTCATTTATTCTGCCATCAGCACATATAGGACCGACTCTTTTGAGGGAAAATCCTCTTTGTATCCTTGTATCTGTAAT
CGGTATATGTCATCTTCTTTTTTTTGGGTTAATTGGTATATGTCATCTTTGTGCATACCATTTTCCTTCTTTTTTTTGTTTC
AGTCAGTTATATTGTATTTGATTATGTCCAAATGTGTATAGGTGATTGGGTCATTACAACAGGTCTGAACATGAAAATCAA
CTTATAGCAGGAATGGCTGTAGAAAGCATCTTTGCACCTTCTCTTTTTCTAATGTATCAGATAGAGTGGCGTTTGTTAAAG
AATATACACTTTGTAAACTTAAAGTAATCATAGTTTCTTTCATGTTCAGTGACAGTTTGTCTCCTCCTCTTGATGGCTGAA
TTTTGTAGTATTATAG
3’ UTR
