# Supplementary figures and images for: A Genome-Wide Analysis of StTGA Genes Reveals the Critical Role in Enhanced Bacterial Wilt Tolerance in Potato During Ralstonia solanacearum Infection
Source: Front Genet. 2022 Jul 26;13:894844. doi: 10.3389/fgene.2022.894844 (PMC9360622; doi:10.3389/fgene.2022.894844)

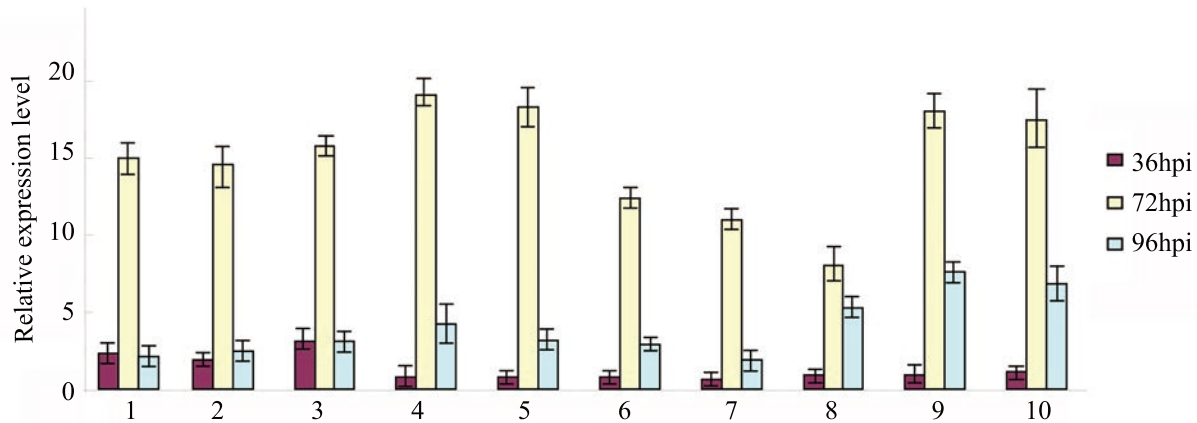

Supplement: Supplementary file 1 [file DataSheet1.zip › Supplementary Additional File S4. Expression of 10 members randomly selected from the StTGA family.pdf]
