# Supplementary material for: A Genome-Wide Analysis of StTGA Genes Reveals the Critical Role in Enhanced Bacterial Wilt Tolerance in Potato During Ralstonia solanacearum Infection
Source: Front Genet. 2022 Jul 26;13:894844. doi: 10.3389/fgene.2022.894844 (PMC9360622; doi:10.3389/fgene.2022.894844)
Supplement: Supplementary file 1 [file Supplementary Additional File S3. The collinear relationship among five species.pdf › Supplementary Additional File S3. The collinear relationship among five species.pdf]

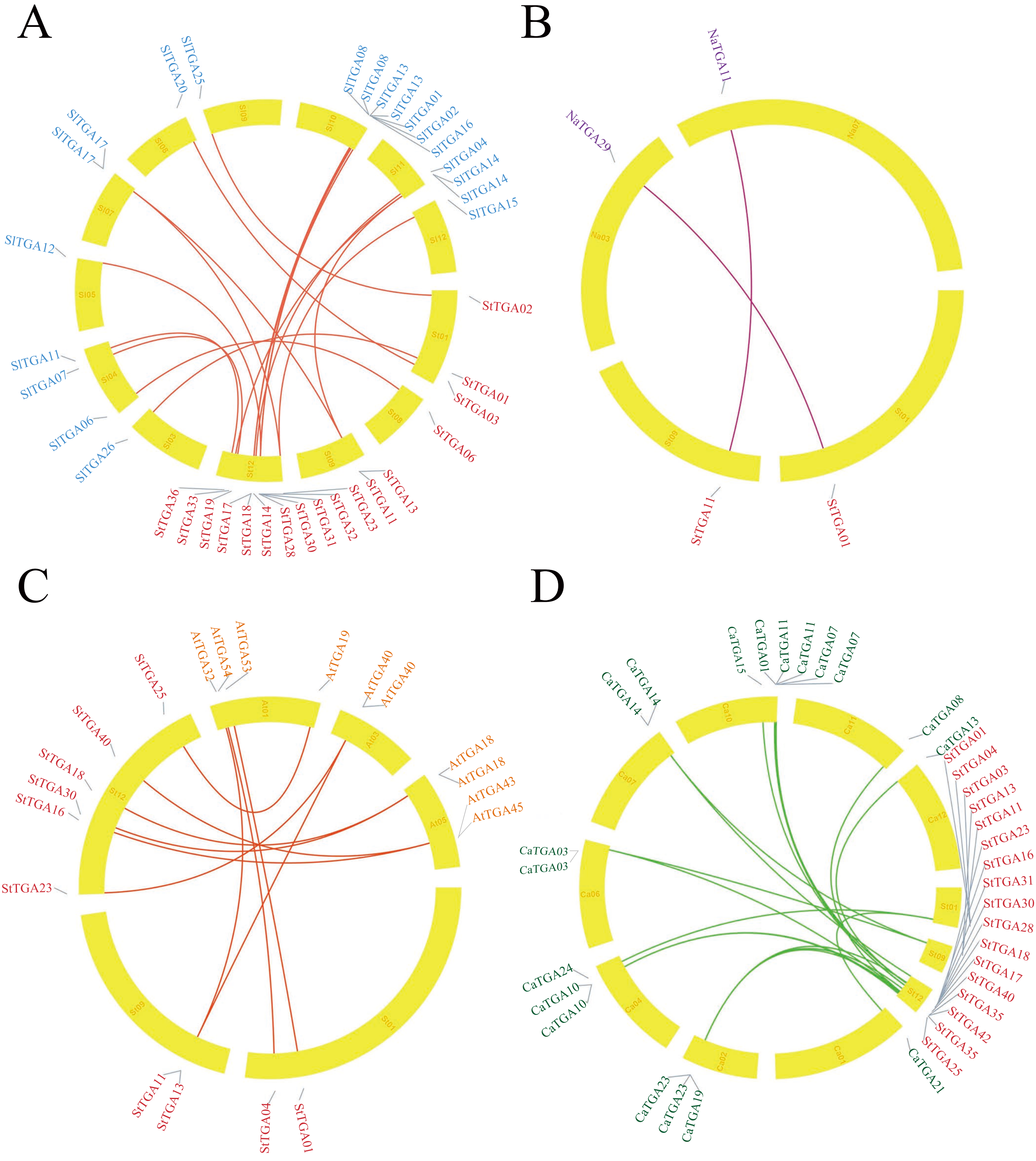
A
SlTGA25
SlTGA20
SlTGA08
SlTGA08
SlTGA13
SlTGA13
SlTGA01
SlTGA02
SlTGA16
SlTGA04
SlTGA14
SlTGA14
SlTGA15
SlTGA17
SlTGA17
SlTGA12
SlTGA11
SlTGA07
SlTGA06
SlTGA26
StTGA02
StTGA01
StTGA03
StTGA06
StTGA36
StTGA33
StTGA19
StTGA17
StTGA18
StTGA14
StTGA28
StTGA30
StTGA31
StTGA32
StTGA23
StTGA11
StTGA13
B
NaTGA11
NaTGA29
StTGA11
StTGA01
C
StTGA25
AtTGA32
AtTGA54
AtTGA53
AtTGA19
AtTGA40
AtTGA40
StTGA40
StTGA18
StTGA30
StTGA16
AtTGA18
AtTGA18
AtTGA43
AtTGA45
StTGA23
StTGA11
StTGA13
StTGA04
StTGA01
D
CaTGA15
CaTGA01
CaTGA11
CaTGA11
CaTGA07
CaTGA07
CaTGA14
CaTGA14
CaTGA08
CaTGA13
StTGA01
StTGA04
StTGA03
StTGA13
StTGA11
StTGA23
StTGA16
StTGA31
StTGA30
StTGA28
StTGA18
StTGA17
StTGA40
StTGA35
StTGA42
StTGA35
StTGA25
CaTGA03
CaTGA03
CaTGA24
CaTGA10
CaTGA10
CaTGA23
CaTGA23
CaTGA19
CaTGA21
